# Genomic characterization of high-risk non-muscle invasive bladder cancer

**DOI:** 10.18632/oncotarget.12661

**Published:** 2016-10-14

**Authors:** Joshua J. Meeks, Benedito A. Carneiro, Sachin G. Pai, Daniel T. Oberlin, Alfred Rademaker, Kyle Fedorchak, Sohail Balasubramanian, Julia Elvin, Nike Beaubier, Francis J. Giles

**Affiliations:** ^1^ Department of Urology, Feinberg School of Medicine, Northwestern University, Chicago, IL, USA; ^2^ Developmental Therapeutics Program, Division of Hematology/Oncology, Feinberg School of Medicine and Robert H. Lurie Comprehensive Cancer Center of Northwestern University, Chicago, IL, USA; ^3^ Department of Pathology, Feinberg School of Medicine, Northwestern University, Chicago, IL, USA; ^4^ Northwestern University Department of Preventive Medicine, Chicago, IL, USA; ^5^ Foundation Medicine Inc, Cambridge, MA, USA

**Keywords:** bladder cancer, mutation burden, progression, invasion

## Abstract

The genetic mechanisms associated with progression of high-risk non-muscle-invasive bladder cancer (HR-NMIBC) have not been described. We conducted selective next-generation sequencing (NGS) of HR-NMIBC and compared the genomic profiles of cancers that responded to intravesical therapy and those that progressed to muscle-invasive or advanced disease. DNA was extracted from paraffin-embedded sections from 25 HR-NMIBCs (22 with T1HG; 3 with TaHG with or without carcinoma *in situ*). Ten patients with HR-NMIBC developed progression (pT2+ or N+) (“progressors”). Fifteen patients had no progression (“non-progressors”). Tissue from 11 patients with metastatic bladder cancer (BC) were analyzed for comparison. We found no difference in frequency of mutations of *TP53*, *PIK3CA*, or *KMT2D* between the primary tumors of progressors compared to non-progressors and metastatic tumors. An increased frequency of deletions of CDKN2A/B was identified in tumors at progression (37%) compared to non-progressors (6%) (*p* = 0.10). We found a significant decrease in total mutational burden (TMB) that has been associated with immunotherapy response comparing non-progressors, progressors and metastatic tumors at 15, 10.1 and 5.1 mutations/MB respectively (*p* = 0.02). This association suggests more advanced tumors have decreased neoantigen burden and may explain the mechanism of BCG response in non-progressors. We found no novel genetic drivers in progressors and HR-NMIBC had many genetic features similar to metastatic BC. Loss of *CDKN2A/B* may occur late during invasion of BC and may represent an important step in progression. Further research is necessary to evaluate TMB and loss of *CDKN2A/B* locus as a biomarker for progression of NMIBC.

## INTRODUCTION

Bladder cancer (BC) is the fourth most common cancer in men [1]. Nearly 80% of patients with BC present with non-muscle-invasive cancers (NMIBC, stage Tis, Ta and T1) and are treated with endoscopic surgical resection, followed by adjuvant intravesical therapies to reduce the risk of recurrence [2, 3]. NMIBCs are clinically heterogeneous with a wide variation in prognosis [4]. Low-grade non-invasive tumors carry a relatively low risk of recurrence (recurrence-free survival [RFS] at 5 years of 20%). Alternatively, T1 high-grade (HG) tumors are associated with RFS of 40% and a 25% mortality rate at 5 years, approaching the prognosis of localized muscle-invasive tumors [5, 6]. Thus, T1HG present a major challenge for clinical and surgical management, especially given the lack of precise molecular-based data to help predict prognosis and enable effective surveillance [7, 8].

Risk stratification of NMIBC is currently based on stage and clinicopathologic parameters. Radical cystectomy is recommended for T1HG cancers with aggressive pathologic features, including carcinoma *in situ*, lymphovascular invasion or variant histology [1, 9]. NMIBCs with these aggressive features are sub-stratified as “high-risk” non-muscle-invasive BC (HR-NMIBC). Despite potentially increased aggressiveness, the majority of patients with HR-NMIBC choose bladder preservation with intravesical immunotherapy such as Bacillus Calmette-Guerin (BCG). Cystectomy, if necessary, is therefore delayed months to years until clinical evidence of recurrence or progression. Compared to immediate cystectomy, the survival of patients treated with delayed cystectomy is reduced [10]. Thus, the lack of precision in the identification of which patients with HR-NMIBCs should be treated with immediate cystectomy reflects a serious challenge in our current therapy paradigm.

The genomic landscape of muscle invasive BC (≥ pT2) was described by the Cancer Genome Atlas project (TCGA). TCGA identified mutations of the *P53 (TP53)/RB1* tumor suppressor pathway in 93% of tumors, activation of the *PTEN/PI3KCA* oncogene pathway in 72%, with frequent loss of function of histone modifying enzymes (MLL2, MLL3, UTX and ARID1a) in 89% [11]. Initially reported in 2014, TCGA did not have long-term clinical data to aid in the analysis of genetic drivers (including drivers associated with progression and survival). Due to the high rate of nucleotide variants in BC (7/MB), identification of true genetic drivers from passenger mutations is challenging, which may be more feasible in lower stage tumors (e.g., T1 cancers) [11]. There currently is very limited genomic data on NMIBC [11], and none on the early “founder” mutations associated with tumor invasion of BC. A comparison of the mutation rate between different stage tumors would facilitate the detection of an association between a relatively high neoantigen burden and response to checkpoint inhibitors as has been demonstrated in melanoma and lung cancers, where response to the immunotherapeutic agents nivolumab and pembrolizumab are directly correlated with total mutational burden (TMB) [12–14]. These questions led to our study of the genetic features of HR-NMIBC. To investigate the genetic drivers of invasion and progression, we performed genetic profiling of HR-NMIBCs that progressed to muscle invasion (“*progressors*”) and compared this profile to cancers of the same stage that responded to conservative therapy (“*non-progressors”*). We also examined the relationship between TMB of HR-NIMBC and progression.

## RESULTS

### Patient characteristics

**Figure 1:**
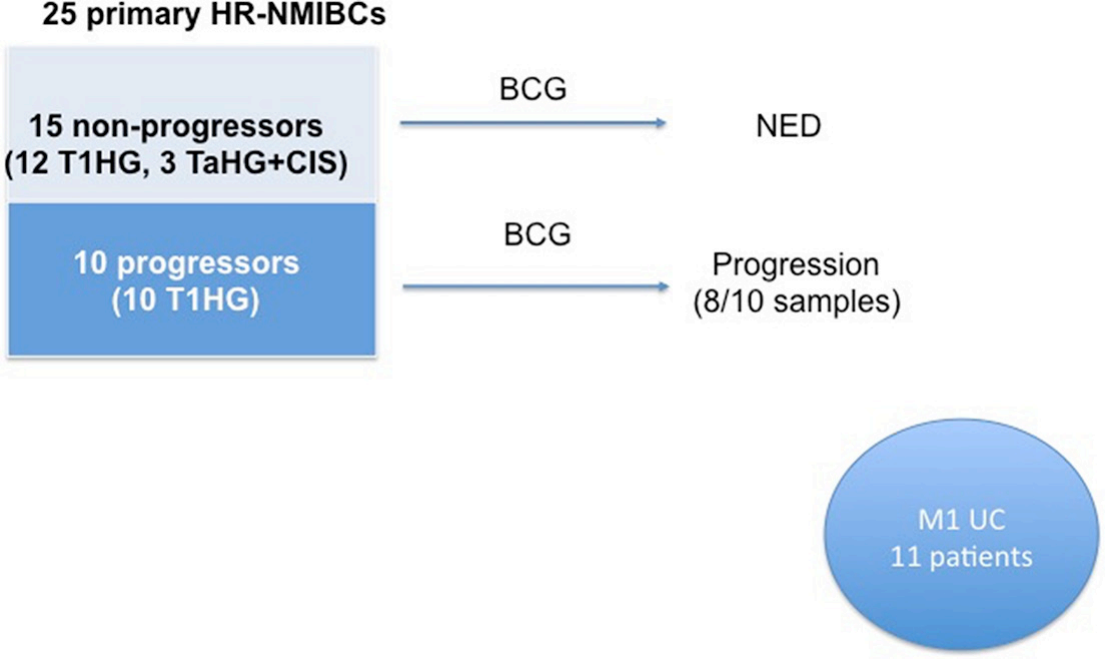
Study schema All patients had high-risk non-muscle invasive bladder cancer. Twenty-five patients were included in the primary cohort. With clinical followup, patients were divided into two groups: those that had progression (10) or those that did not progress or recur (15). Of the progressors, eight patients had tissue sufficient for genomic profiling. This cohort was compared to 11 patients with metastatic urothelial carcinoma.

A total of 25 patients with an initial diagnosis of HR-NMIBC were identified Figure 1. These patients underwent TURBT and most of them received standard of care adjuvant treatment with intravesical BCG or a combination of BCG and interferon-alfa. Patients were then followed with surveillance cystoscopies every 3 months. Fifteen patients had no progression at a median follow up of 53 months (Table 1). Ten patients with T1HG were initially treated with BCG but had recurrence with locally advanced (pT2+) or metastatic (N+) cancer (Table 2). This group of patients was identified as “progressors” and the median time from diagnosis to progression was 9 months. Of the 10 patients with clinical and pathologic progression, 8 patients had tumors with sufficient tissue to allow genomic profiling at the time of progression, while the remaining two patients had insufficient tissue to allow genetic analysis. For comparison, a third group consisting of 11 patients with metastatic BC was also included (Table 3).

**Table 1:** Clinical characteristics of patients without progression

Case No	Age	Gender	Race	Stage at baseline	Follow up (months)
1	91	Male	W	T1HG	58
2	90	Male	W	T1HG	45
3	61	Female	W	T1 HG+CIS	67
4	74	Male	W	T1HG	49
5	81	Male	O	TaHG+CIS	60
6	50	Female	W	T1HG	36
7	76	Male	W	T1HG	38
8	71	Male	W	T1HG+CIS	62
9	67	Male	W	TaHG	72
10	59	Male	AA	TaHG	81
11	80	Male	AA	T1HG,+CIS	40
12	80	Male	AA	T1HG	28
13	49	Male	W	T1HG+CIS	75
14	59	Male	W	T1HG	53
15	49	Male	H	T1HG	23

**Table 2:** Clinical characteristics of patients with progression to muscle-invasive disease

Case No	Age	Gender	Race	Stage at baseline	Stage at progression	Time to Progression (months)
16	65	Male	AA	T1HG	cT1NxM1 (bone)	8
17	72	Male	W	T1HG	cT2NxMx	4
18	61	Male	AA	T1HG	pT1N1Mx	3
19	60	Male	W	T1HG	pT4N0Mx	41
20	81	Male	W	T1HG	cT2N+M+	30
21	69	Male	W	T1HG	pT4aN0Mx	6
22	88	Male	W	T1HG	cT2NxM1 (liver)	10
23	86	Male	W	T1HG	cT4aNxMx	23
24	68	Male	W	T1HG	pT4aN0	16
25	59	Male	W	T1HG	pT3N2Mx	3

**Table 3:** Clinical characteristics of patients with metastatic disease

Case No	Age	Gender	Race	Metastatic sites
26	72	Female	AA	LN
27	84	Male	O	LN, lung, brain
28	83	Male	W	LN, lung
29	75	Male	O	LN
30	75	Male	W	LN, bone
31	64	Female	O	LN, liver, lung
32	49	Male	O	LN, liver
33	75	Male	O	LN
34	61	Male	O	LN
35	81	Female	O	LN, bone, lung
36	59	Male	W	Lung, pleura, liver, bone

### Total mutational burden

The TMB differed between non-progressors versus progressors. Non-progressors had the highest TMB at 15.0 mutations/MB compared to a rate of 12.8 mutations/MB for the initial tumors of the progressors (i.e. samples collected at the time of initial diagnosis). The TMB of progressors at recurrence was lower (10.1 mutations/MB) compared to their TMB at presentation (12.8 mutations/MB). Metastatic BC had the lowest TMB with a mutation rate of 5.1 mutations/MB. Thus, there was a statistically higher rate of variants in tumors that responded to intravesical therapy with a mutational rate of approximately one third (15/MB) of that in tumors that progressed and one-half in metastatic cancers (5.1/MB)(*p* = 0.02).

### Comparison of primary tumors: progressors and non-progressors

**Figure 2:**
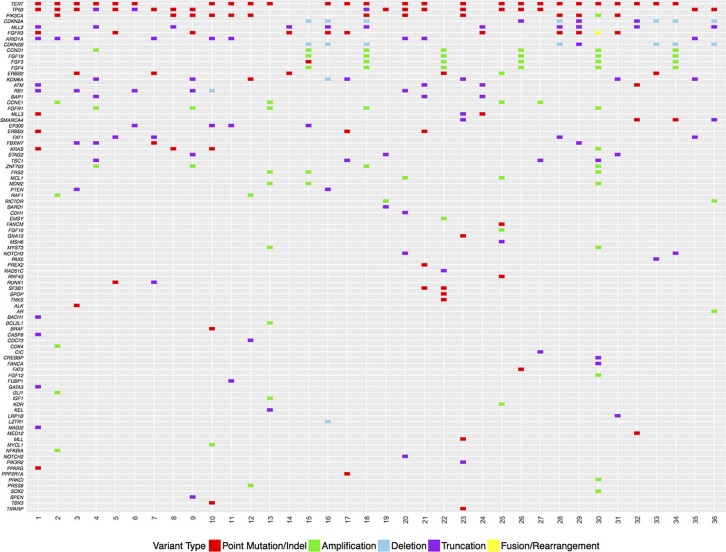
Genetic features of primary tumors from non-progressors, progressors and patients with metastatic urothelial carcinoma Type of mutation is listed below the plot. Genes are listed by most commonly mutated to least most commonly mutated across the entire cohort. Cases correspond to patients in Tables 1–3. Tumors 1–15 are high-risk patients with no progression (see Table 1). Tumors 16–25 are the primary tumors that later had progression (see Figure 3) and are well described clinically in Table 2. Tumors 26–36 are tumors from patients that are metastatic and are described clinically in Table 3.

**Figure 3:**
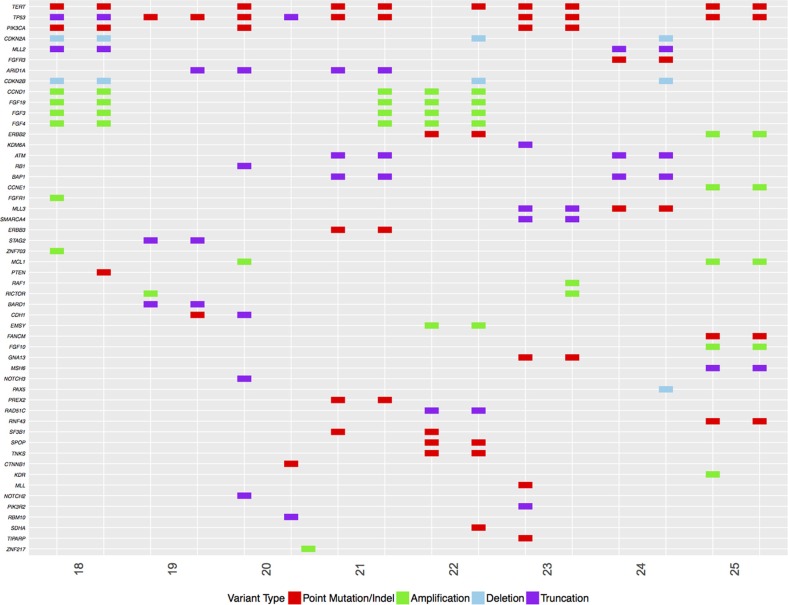
Comparison of genetic features of the same tumors at presentation versus features at progression Each tumor is listed with original tumor (left) and tumor at progression on the right. Type of mutation is listed below the plot. The clinical features of each patient are summarized in Table 2.

We compared the genomic features of tumors at the time of initial resection between the progressors and non-progressors (Figures 2 and 3; Table 4). No specific genetic features distinguished progressors from non-progressors at the time of diagnosis. The highest frequency variant in all samples was mutation of the promoter region of *TERT* with an identified frequency of 70% in progressors, 66% in non-progressors (*p* = 0.99), and 82% among patients with metastatic disease. Mutations in *TP53* and *RB1* were present in 60% and 10% of progressors and 60% and 33% of non-progressors, respectively. The frequency of *PIK3CA* and *PTEN* mutations was 30% and 10% in progressors and 40% and 6% of non-progressors, respectively. Mutations in *KMT2D* (MLL2) were found in 30% of progressors and 26% of non-progressors while mutations of *ARID1A* were found in 20% of progressors and 40% of non-progressors.

**Table 4:** Incidence of genomic alterations in relevant genes potentially associated with risk of progression to muscle-invasive disease

Genes	Non-progressors (group 1)	Progressors baseline (group 2)	Progressors muscle-invasive disease (group 3)	Metastatic group (group 4)	Group 1 vs 2 (*P* value)	Group 2 vs 3 (*P* value)	Group 3 vs 4 (*P* value)
*TERT*	10/15 (66%)	7/10 (70%)	5/8 (62%)	9/11 (81%)	0.99	0.99	0.60
*TP53*	9/15 (60%)	6/10 (60%)	6/8 (75%)	4/11 (36%)	1.00	0.99	0.17
*RB1*	5/15 (33%)	1/10 (10%)	0/8 (0%)	0/11 (0%)	0.34	0.32	1.00
*PIK3CA*	6/15 (40%)	3/10 (30%)	2/8 (25%)	4/11 (36%)	0.69	0.32	0.99
*PTEN*	1/15 (6%)	1/10 (10%)	1/8 (12%)	0/11 (0%)	0.99	0.32	0.42
*KMT2D (MLL2)*	4/15 (26%)	3/10 (30%)	2/8 (11%)	4/11 (36%)	0.99	0.99	0.99
*ARID1A*	6/15 (40%)	2/10 (20%)	2/8 (25%)	1/11 (9%)	0.40	0.99	0.55
*CDKN2A/B*	1/15 (6%)	2/10 (20%)	3/8 (37%)	7/11 (63%)	0.54	0.16	0.37
*CCND1 amp*	2/15 (12%)	2/10 (20%)	3/8 (37%)	3/11 (27%)	0.99	0.32	0.99
*FGFR/FGF*	9/15 (60%)	6/10 (60%)	5/8 (62%)	6/11 (54%)	1.00	0.32	0.99

### Novel mutations at progression

To identify the genomic features associated with progression of NMIBC tumors, we further evaluated the eight tumors that progressed and had sufficient tissue available for complete genomic profiling and compared the results to the genomic profile of the corresponding primary tumor (Figure 3). *CCND1* was amplified in 2/10 progressors at baseline and 3/8 at progression, 3/11 metastatic tumors and only 2/15 non-progressors. Deletion of *CDKN2A/B* was identified in 3/8 progressors: one was present in both the initial and post-progression sample, and the other two acquired loss of *CDKN2A/B* between diagnosis and progression. In non-progressors, *CDKN2A* and *CDKN2B* mutations were only found in 1 patient (6%) and this patient was wild type for *TP53* and *RB1.* Overall, comparison of 7/8 tumors at progression identified de novo mutations compared to their primary (Figure 1). These mutations included *PTEN*, *ARID1A*, *CDH1, SDHA* and *RAF1*. Amplification of a cluster of genes located at 11q13 including *FGF3*, *FGF4*, *FGF19* and *CCND1* was acquired in one patient. This 11q13 gene cluster was also amplified in the primary and progression tumors of two more NMIBC cancers. Overall, this amplification cluster was only present in progressors (38%) and not present in non-progressors. This amplification was also present in 27% of metastatic patients.

Seven of eight tumor samples retained the genetic features of the primary tumor suggesting they were recurrence of the same clonal cancer. One progressor was noted to have a significantly different mutation profile at the time of progression compared to presentation with almost no overlap of mutations (Case #23). This patient originally presented with a NMIBC tumor, subsequently developed a tumor on the opposite side of the bladder that was muscle-invasive 30 months later. Haplotype analysis confirmed that both specimens originated from the same patient, but he was found to have a germline proofreading mutation in the *POLE* gene that is involved in DNA repair and chromosomal DNA replication.

## DISCUSSION

The management of HR-NMIBC remains challenging due to limitations in staging and molecular biomarkers to predict response to BCG or progression to muscle-invasive disease. While 90% of HR-NMIBC patients are treated with a bladder preservation strategy, up to 25% will die of BC at 5 years [5]. Various clinical and pathologic features have been proposed to risk-stratify patients with high vs. “very high” risk patients, with these features including lymphovascular invasion, variant histology and the presence of CIS [7]. While all T1HG tumors demonstrate lamina propria invasion, at least 30–40% will respond to BCG and have no progression while those patients that have a radical cystectomy have a 40% risk of upstaging to muscle-invasive disease and a 10% risk of lymph node involvement. Thus, the physician must make a judgment call with limited information on the responsiveness of a high-risk tumor to BCG, with potentially detrimental consequences to prognosis if the tumor does not respond. Alternatively, avoiding the potential morbidities of a radical cystectomy for those patients with a low risk of disease progression would be very beneficial.

This study aimed to identify novel genetic features of HR-NMIBC that may be associated with progression to muscle-invasion. We hypothesized that HR-NMIBC may have unique genetic events driving the pathway to progression, and perhaps share a similar genomic landscape with tumors of advanced stage, such as those described in the TCGA. Genomic profile analysis showed a similar frequency of mutations in *TERT, TP53, RB1, PTEN, PIK3CA, KMT2D* and *ARID1A* at presentation among progressors and non-progressors. We identified a non-significant association of increased frequency of loss of *CDKN2A/B* in progressors that was absent in non-progressors. Importantly, the non-progressor that had a *CDKN2A* mutation was wild type for *TP53*. These data support the thesis that the p16/P53/RB1 signaling axis contributes to progression of HR-NMIBC. By comparison of two separate tumors from the initial treatment to progression, we confirmed the clonal expansion of invasive bladder cancer. In this limited cohort, no single gene emerged as a potential biomarker to identify patients who progressed from those who did not. In contrast, TMB was significantly lower amongst the tumors that progressed to muscle-invasion and metastasized compared to those which responded to intravesical BCG and did not progress. In other tumors, including melanoma and lung cancers, response to the immunotherapeutic agents nivolumab and pembrolizumab were associated with higher TMB [15–17]. Thus, the relationship between high TMB and bladder tumors that do not progress raises the possibility that intravesical BCG may also be more effective in cancers with higher neoantigen burden. Conversely, metastatic tumors and tumors that had progression had lower TMB making them potentially more resistant to immunotherapy, including BCG. In a recently published trial of advanced bladder cancer, treatment with atezolizumab (anti-PD-L1 antibody) resulted in objective response rates of 26% (95% CI 18–36) in tumors with ≥ 5% PD-L1 expressing infiltrating immune cells [17]. The median TMB was significantly increased in responders compared with non-responders, however the association between mutation load and response was unrelated to TCGA subtype or immune cell subgroup. Further research is necessary to evaluate if the neoantigen burden can predict response to intravesical immunotherapy or function as an independent prognostic variable in this patient group [18, 19].

Similar to findings described in TCGA, we found a high frequency of *TP53* mutations in both progressors and non-progressors [11]. While the TCGA described a frequency of 49%, we found a high rate of *TP53* mutation of 57–60% in both cohorts. This data suggest that loss of P53 is not a checkpoint for progression or a biomarker of aggressive cancer. Loss of P53 was hypothesized to occur as a late event in progression of bladder cancer. Our data on the temporal progression of HR-NMIBC, suggests that P53 loss occurs early, compared to loss of P16. As most of these tumors had invasion (22/25), loss of P53 may be an early event in the invasive process. These data confirms the lack of prognostic strength of P53 as a biomarker in bladder cancer and may explain why this has not been effective to guide therapy [20, 21].

A limitation of this study was the number of patients included in our analysis. While we were only able to fully compare tumors at diagnosis and progression in eight patients, this is the largest series to date evaluating HR-NMIBC with close clinical follow-up. Genetic analysis of tumors in progessors was limited to tumors that we could obtain DNA (8/10 patients) and this could result in bias of genetic mutations found in larger size tumors. Our genetic analysis was limited to 315 genes and other genes that were not included could serve as drivers of urothelial malignancy. We did not perform a validation of our analysis as there are no publically available data sets of high-risk non-muscle invasive bladder tumors that have genetic information with mutation burden. Future prospective studies of HR-NMIBC involving whole genome sequence analysis may identify additional candidate driver mutations in early stage bladder cancer.

## CONCLUSIONS

Genetic analysis of HR-NMIBC identified genetic similarity between tumors that recur and progress compared to tumors that respond to endoscopic resection and BCG. Tumors that respond to intravesical therapy have a higher rate of mutation compared to tumors that progress. The loss of *CDKN2A/B* occurred in 38% of patients that progressed to muscle-invasion, suggesting its potential role as an important event during invasion. Future studies are necessary to evaluate its role as a biomarker and identify the role of neoantigen burden in HR-NMIBC.

## MATERIALS AND METHODS

### Patient identification

Following IRB approval, the Northwestern Medicine Enterprise Data Warehouse, a comprehensive and integrated repository of clinical and research data, was used to identify patients with HR-NMIBC [22]. From this analysis, patients were assigned to two cohorts: 1) 15 patients with pathologically identified HR-NMIBC (T1HG or TaHG with CIS) that had no progression over ≥ two years and 2) 10 patients with HR-NMIBC who progressed to muscle-invasive BC despite receiving complete resection followed by intravesical immunotherapy. For both cohorts, all pathologic specimens had muscle present in the specimen and all patients underwent a second transurethral resection within 8 weeks of the first operation and no tumor was identified on repeat transurethral resection of bladder tumor (TURBT). All patients but one received a minimum 6-week course of intravesical immunotherapy and had at least 2 years of follow-up with cystoscopic investigation without visual evidence of disease recurrence. Progression in the “progressor” cohort had pathologic muscle invasion by cystectomy, TURBT, lymph node biopsy, or biopsy-demonstrated visceral metastasis. A third cohort of 11 patients with metastatic bladder cancer was included as a comparison cohort representing advanced disease that was treated in the same time as patients in the two NMIBC cohorts. Three patients in the progressor cohort received a second induction course of BCG for superficial recurrence. All eligible specimens were re-reviewed by a genitourinary pathologist to confirm the diagnosis and stage prior to study inclusion. Given the well-described poor prognosis of the micropapillary subtype of urothelial carcinoma, this histology was excluded from this study as well as other histologic variants.

### Extraction of DNA and next-generation sequencing (NGS)

Once the archived specimens were identified, ten unstained, de-identified slides (4 microns thickness) were submitted for comprehensive genomic profiling at Foundation Medicine. Hybridization capture of 3,769 exons of 315 cancer-related genes plus introns from 28 genes commonly rearranged in cancer (FoundationOne^®^) was applied to ≥ 50ng of DNA extracted from archival formalin-fixed, paraffin embedded bladder tumor tissue and sequenced to high, uniform coverage [23]. All classes of genomic alterations (base substitutions, small insertions and deletions (indels), rearrangements, copy number alterations) were determined. TMB was calculated by counting all synonymous and nonsynonymous variants as well as indels across a 1.25 megabase coding region spanning 315 genes. Germline polymorphisms were filtered by comparing dbSNP, ExAC as well as internal FMI databases, in addition to using a proprietary somatic/germline algorithm (SGZ). Due to the high mutation rate associated with oncogenes and tumor suppressors, these genes were removed to limit biasing in the calculation of TMB[24].

### Statistical analysis

TMB was compared between groups using the Wilcoxon rank sum test. Mutation rates were compared between groups of different patients using Fisher's exact test. Mutation rates were compared between progressors at baseline and at progression using McNemar's test. For analysis, mutation data were available for 15 non-progressors, for all 10 progressors at baseline but only for 8 at progression, and for all 11 patients with metastatic disease.
